# AISI, SIRI, and MLR in Predicting Surgical Outcomes After Radical Cystectomy: Revisiting Inflammatory Risk Markers

**DOI:** 10.3390/medicina61101756

**Published:** 2025-09-27

**Authors:** Mertcan Dama, Enis Mert Yorulmaz, Serkan Özcan, Osman Köse, Sacit Nuri Görgel, Yiğit Akın

**Affiliations:** 1 Ministry of Health Izmir City Hospital, 35540 Izmir, Türkiye; 2 Department of Urology, Izmir Katip Celebi University, 35620 Izmir, Türkiye; enismertyorulmaz@yahoo.com (E.M.Y.); drserkanozcan@hotmail.com (S.Ö.); oskose@gmail.com (O.K.); sngorgel@hotmail.com (S.N.G.); yigitakin@yahoo.com (Y.A.)

**Keywords:** radical cystectomy, systemic inflammatory response index, postoperative complications, AISI, MLR

## Abstract

*Background and Objectives*: This study aimed to evaluate the predictive value of systemic inflammatory response markers—namely, the Systemic Inflammatory Response Index (SIRI), Aggregate Index of Systemic Inflammation (AISI), and Monocyte-to-Lymphocyte Ratio (MLR)—in determining the occurrence of major complications following radical cystectomy. *Materials and Methods*: A retrospective analysis was conducted on 200 patients who underwent open radical cystectomy with ileal conduit diversion. Demographic, clinical, and laboratory variables, including albumin, creatinine, eGFR, smoking, and ASA score, were collected. SIRI, AISI, and MLR were calculated from preoperative blood counts. Major complications and their subtypes (infectious, wound, cardiopulmonary, thrombotic, and anastomotic) were adjudicated independently. Statistical analyses included multivariable logistic regression, ROC curves, calibration (Hosmer–Lemeshow, intercept, slope, and plots), bootstrap resampling (B = 2000), linearity checks (restricted cubic splines and Box–Tidwell), incremental value metrics (ΔAUC, IDI, and NRI), and decision-curve analysis (DCA). *Results:* Major complications occurred in 57 patients (28.5%). SIRI values were significantly higher in patients with major complications (median 2.12 vs. 1.63, *p* = 0.006), whereas AISI and MLR did not differ. SIRI remained an independent predictor in multivariable analysis (OR 1.37, 95% CI 1.01–1.86, *p* = 0.045). An AUC of 0.624 (95% CI 0.538–0.709) with a negative predictive value of 83.3% was observed for SIRI. The baseline clinical model yielded an AUC of 0.648, and an AUC of 0.672 was obtained when SIRI was added (ΔAUC = +0.024, 95% CI −0.022–0.071, *p* = 0.16). Calibration was excellent (intercept = 0.07, slope = 1.08), and superior net benefit was demonstrated for the SIRI-augmented model within threshold probabilities of 0.15–0.45 in DCA. A statistically significant improvement in IDI (0.024, *p* = 0.024) was identified, while NRI was positive but not significant. Subtype analyses indicated that the strongest associations of SIRI were with infectious and wound complications. *Conclusions:* SIRI was found to be an independent predictor of major complications after open radical cystectomy. Although gains in discrimination were modest, incremental analyses demonstrated improved calibration and net clinical benefit when SIRI was incorporated into a clinical model. External validation is required before translation into clinical practice.

## 1. Introduction

Bladder cancer remains a significant global health concern, with over 573,000 new cases annually, and muscle-invasive bladder cancer (MIBC) constitutes approximately 25% of these diagnoses [1]. For these patients, radical cystectomy (RC) with pelvic lymph node dissection remains the cornerstone of curative treatment, as endorsed by the European Association of Urology guidelines [2]. Despite advancements in surgical techniques and perioperative care, RC continues to be associated with substantial morbidity, with contemporary series reporting complication rates ranging from 30% to 64% [3,4]. These postoperative complications not only impair patient recovery and quality of life but also impose a significant burden on healthcare systems [3], highlighting the critical need for reliable preoperative risk stratification tools to optimize patient selection, guide counseling, and tailor enhanced recovery protocols.

A key factor modulating surgical outcomes is the patient’s systemic inflammatory state, which has been increasingly recognized as a driver of adverse postoperative events [5]. Preoperative inflammation disrupts immune homeostasis, leading to impaired wound healing, increased susceptibility to infections, and dysregulated stress responses [6]. This has spurred interest in easily measurable inflammatory biomarkers, such as the neutrophil-to-lymphocyte ratio (NLR) and platelet-to-lymphocyte ratio (PLR), which have demonstrated prognostic value in various oncologic and surgical settings [7]. However, these markers provide only a partial representation of the inflammatory cascade, prompting the exploration of more comprehensive indices.

More recently, composite inflammatory indices, such as the Systemic Inflammation Response Index (SIRI), Aggregate Index of Systemic Inflammation (AISI), and Monocyte-to-Lymphocyte Ratio (MLR), have emerged as promising tools. These indices integrate multiple leukocyte subsets and platelets, offering a more holistic assessment of systemic inflammation [8]. While SIRI and AISI have shown prognostic utility in gastrointestinal and other malignancies [9], their role in predicting postoperative complications following RC—a procedure with uniquely high inflammatory and physiologic stress—remains unexplored [10]. Given the invasive nature of RC and its substantial complication profile, elucidating the predictive capacity of these indices could fill a critical gap in perioperative risk assessment.

The primary objective of this study was to evaluate the predictive value of preoperative SIRI, AISI, and MLR for major complications following RC. We hypothesized that SIRI, as a composite marker of neutrophil, monocyte, and lymphocyte interactions, would serve as an independent predictor of adverse postoperative outcomes, offering a more robust stratification tool than conventional biomarkers. By addressing this knowledge gap, our findings may contribute to improved preoperative risk assessment, personalized patient counseling, and targeted interventions to mitigate postoperative morbidity.

## 2. Materials and Methods

This retrospective observational study was conducted at a tertiary academic urology center and included 200 consecutive patients who underwent open radical cystectomy for muscle-invasive bladder cancer between January 2016 and December 2023. The study received approval from the Institutional Review Board. Given the retrospective design of this study and the use of anonymized data from existing medical records, the requirement for written informed consent was waived by the institutional ethics committee.

Patients were eligible for inclusion if they had histologically confirmed pure urothelial carcinoma staged ≥T2 on transurethral resection, no evidence of distant metastases on preoperative imaging (CT/MRI of abdomen/pelvis and chest CT), and underwent curative-intent open RC with pelvic lymph node dissection. In all cases, urinary diversion was achieved via ileal conduit formation (Bricker procedure). Other forms of urinary diversion, such as continent reservoirs or neobladders, were not utilised during the study period, ensuring procedural homogeneity in terms of surgical complexity and associated risk profiles. We excluded patients with non-urothelial histologies, metastatic disease at surgery, prior pelvic radiation or major pelvic surgery, chronic inflammatory conditions, immunosuppressant use (including corticosteroids within 3 months preoperatively), incomplete laboratory data, or concurrent active malignancies (except non-melanoma skin cancer). The exclusion of patients receiving neoadjuvant therapy was intentional to eliminate potential confounding effects on inflammatory markers. As per institutional practice, patients with clinical or laboratory evidence of acute infection (e.g., fever, positive cultures, recent antibiotic therapy, leukocytosis/CRP elevation) are not scheduled for elective radical cystectomy until resolution. Therefore, no patient with acute infection was included in this study, and explicit exclusion criteria were not required.

Data collection encompassed demographic variables (age, sex, BMI), comorbidities (diabetes mellitus, hypertension, coronary artery disease confirmed through ICD-10 codes or active medications) and operative parameters (operative time, length of stay, intraoperative transfusion status). Preoperative complete blood count results, obtained within 7 days before surgery (92% within 48 h), were used to calculate three inflammatory indices: the Systemic Inflammation Response Index (SIRI; neutrophils × monocytes/lymphocytes), Aggregate Index of Systemic Inflammation (AISI; neutrophils × monocytes × platelets/lymphocytes), and Monocyte-to-Lymphocyte Ratio (MLR; monocytes/lymphocytes). Extreme values were retained after verifying the absence of acute infection. In addition, preoperative serum albumin, creatinine, and estimated glomerular filtration rate (eGFR) values, as well as smoking status and ASA score, were recorded. Postoperative complications were further categorized into subtypes (infectious, wound-related, cardiopulmonary, thrombotic, and anastomotic) to enable exploratory analyses.

The primary outcome focused on major postoperative complications (Clavien-Dindo classification ≥IIIa) occurring within 30 days postoperatively, capturing both in-hospital and post-discharge events. Post-discharge events were captured through hospital readmission records and follow-up visits documented in the electronic medical record. Two urologists, blinded to inflammatory marker values, independently adjudicated all complications.

Statistical analyses were performed using Jamovi v2.6 (The Jamovi Project, Sydney, Australia). Continuous variables with non-normal distributions (confirmed by Shapiro–Wilk tests) were reported as medians with interquartile ranges. Comparative analyses employed Mann–Whitney U tests for inflammatory markers and chi-square or Fisher’s exact tests for categorical variables. Multivariable logistic regression included pre-specified clinically relevant covariates (age, BMI, hypertension, diabetes, coronary artery disease, and SIRI), irrespective of univariate associations. Multicollinearity was assessed using variance inflation factors (all < 1.10).

SIRI was analysed as a continuous variable in regression and model performance analyses. ROC analysis with the DeLong method evaluated discrimination, and the Youden index was used to determine an optimal cut-off value, which was reported only for descriptive purposes. Model performance was further assessed using AUC with 95% confidence intervals, calibration by Hosmer–Lemeshow test, calibration-in-the-large (intercept and slope), calibration plots, and internal validation with bootstrap resampling (B = 2000). The functional form of SIRI was examined using restricted cubic splines (df = 3) and the Box–Tidwell test to confirm linearity. Incremental value was quantified using ΔAUC, integrated discrimination improvement (IDI), and net reclassification improvement (NRI), while clinical utility was assessed by decision-curve analysis (DCA). All advanced analyses were conducted in R (version 4.3.0, R Foundation for Statistical Computing, Vienna, Austria) using established packages (pROC, boot, PredictABEL, and rmda). All statistical tests were two-sided with significance set at *p* < 0.05.

## 3. Results

A total of 200 patients who underwent radical cystectomy were included in the study. The mean age was 67.8 ± 7.1 years, and the mean BMI was 26.0 ± 4.3 kg/m^2^. Among the patients, 57 (28.5%) experienced Clavien-Dindo grade ≥ IIIa major complications within 30 days postoperatively. Smoking status, serum albumin, creatinine, and eGFR did not differ significantly between patients with and without major complications. The clinical and demographic characteristics of the patients are presented in Table 1.

**Figure 1:**
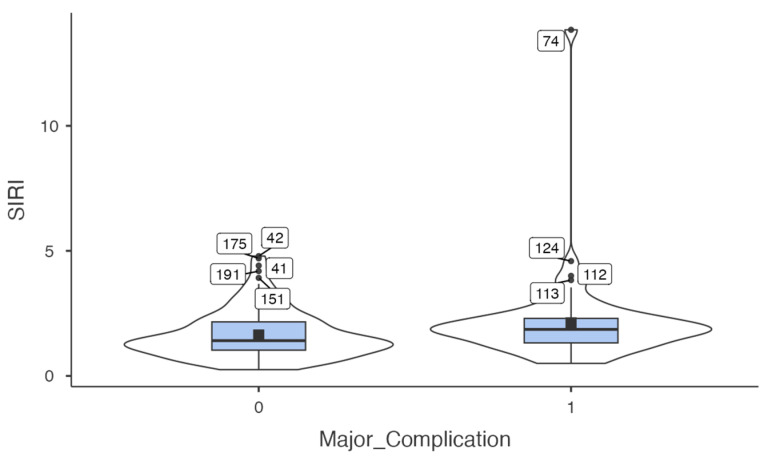
Violin plot showing the distribution of SIRI values in patients with and without major postoperative complications.

Among the inflammatory indices, SIRI was significantly higher in patients with major complications compared to those without. AISI and MLR did not differ significantly between the groups. Table 2 summarises the distribution of preoperative inflammatory indices (SIRI, AISI, MLR) according to the occurrence of major complications. To further illustrate this difference, Figure 1 presents a violin plot demonstrating the distribution of SIRI values according to complication status, highlighting both the elevated median values and greater variability observed in patients with major complications.

When complications were analysed by subtype, preoperative SIRI was significantly higher in patients who developed infectious complications compared with those who did not (median 1.91 vs. 1.41, *p* = 0.004), and borderline higher in patients with wound-related complications (median 1.87 vs. 1.49, *p* = 0.046). No significant differences in SIRI values were observed for cardiopulmonary, thrombotic, or anastomotic complications (Table 3).

**Figure 2:**
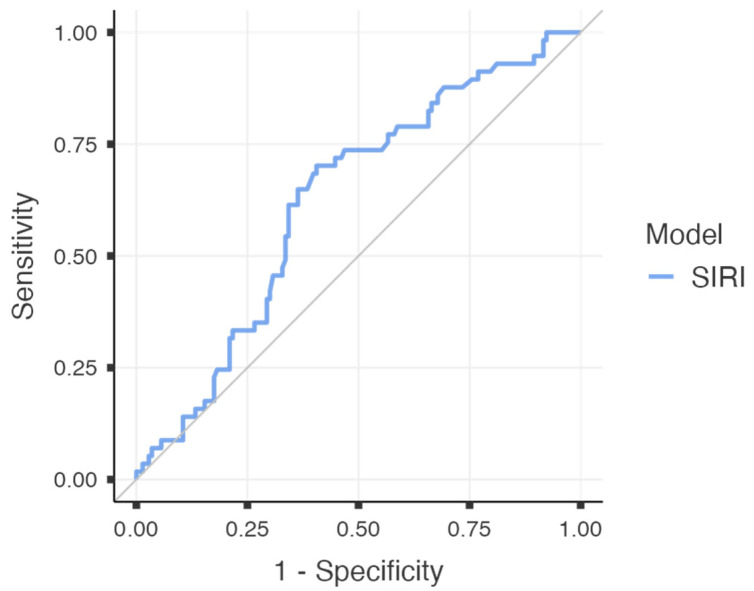
ROC curve for SIRI predicting major complications after radical cystectomy (AUC:0.624).

To further evaluate the predictive performance of SIRI for major postoperative complications, a receiver operating characteristic (ROC) curve analysis was performed. The area under the curve (AUC) was calculated as 0.624 (95% CI: 0.538–0.709; *p* = 0.003), indicating a fair discriminatory capacity. At an optimal cut-off value of 1.545, SIRI demonstrated a sensitivity of 70.2%, specificity of 59.4%, positive predictive value (PPV) of 40.8%, and a notably high negative predictive value (NPV) of 83.3%. The overall diagnostic accuracy was calculated as 62.5%. The corresponding ROC curve is illustrated in Figure 2, and the diagnostic metrics are summarised in Table 4.

All relevant clinical and demographic variables were included in the multivariate logistic regression model to identify independent predictors of major complications. SIRI remained an independent predictor of major complications (OR = 1.37, 95% CI: 1.01–1.86, *p* = 0.045), while diabetes mellitus showed borderline significance (OR = 1.89, 95% CI: 0.95–3.86, *p* = 0.07). Table 5 presents the results of the multivariate logistic regression analysis, evaluating the independent associations between clinical variables and the occurrence of major postoperative complications.

**Figure 3:**
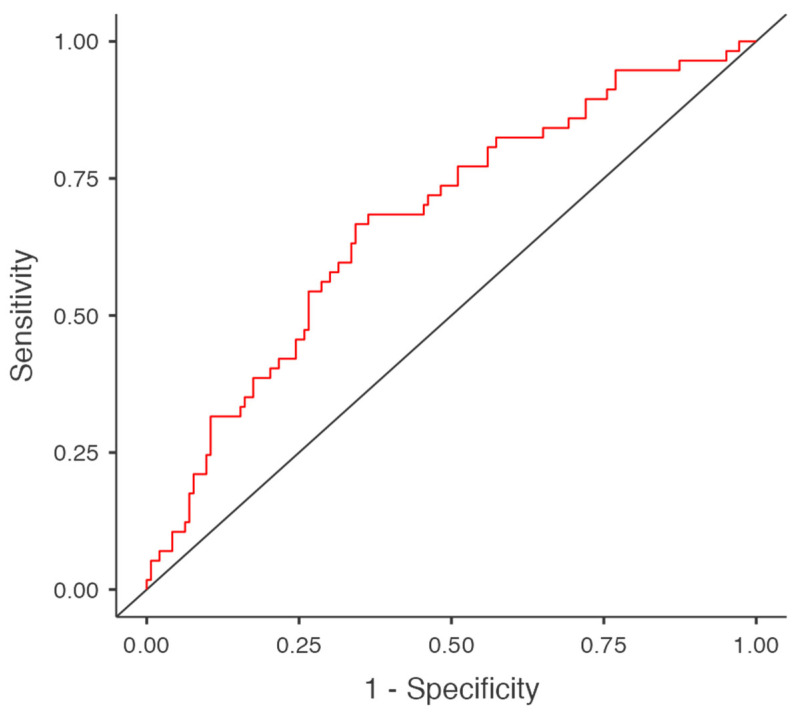
ROC curve demonstrating the predictive performance of the multivariate logistic regression model for major complications after radical cystectomy (AUC = 0.676). The red line represents the ROC curve, while the grey diagonal line indicates the reference line (AUC = 0.5), corresponding to no discriminative ability.

To further assess the predictive performance of the multivariate logistic regression model, a receiver operating characteristic (ROC) curve was constructed. The area under the curve (AUC) was calculated as 0.676, indicating fair discriminative capacity for predicting major postoperative complications. This suggests that the combined model—including SIRI and clinical variables—offers improved prognostic performance compared to SIRI alone. The overall diagnostic performance of the multivariate model is illustrated in Figure 3.

The baseline clinical model (age, BMI, hypertension, diabetes mellitus, and coronary artery disease) yielded an AUC of 0.648 (95% CI 0.560–0.735) with acceptable calibration (Hosmer–Lemeshow χ^2^ = 10.41, df = 8, *p* = 0.236). SIRI alone demonstrated modest discrimination (AUC = 0.624, 95% CI 0.538–0.709). Adding SIRI to the baseline model improved the AUC to 0.672 (95% CI 0.586–0.752), corresponding to a ΔAUC of +0.024 (95% CI −0.022 to +0.071, *p* = 0.16). Although this increase did not reach statistical significance, calibration was markedly improved (Hosmer–Lemeshow χ^2^ = 3.33, df = 8, *p* = 0.910).

**Figure 4:**
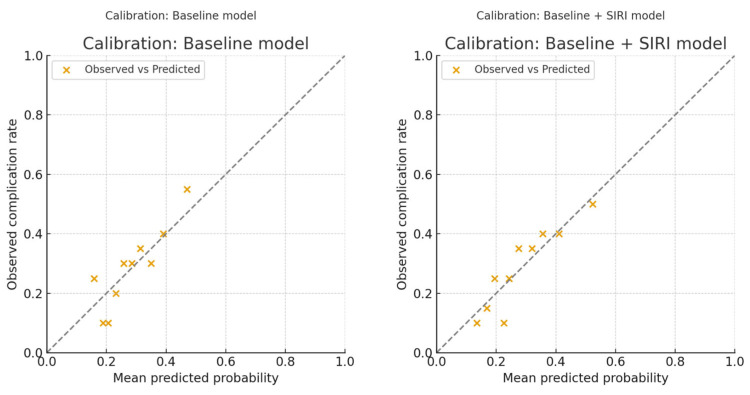
Calibration plots of predictive models.

**Figure 5:**
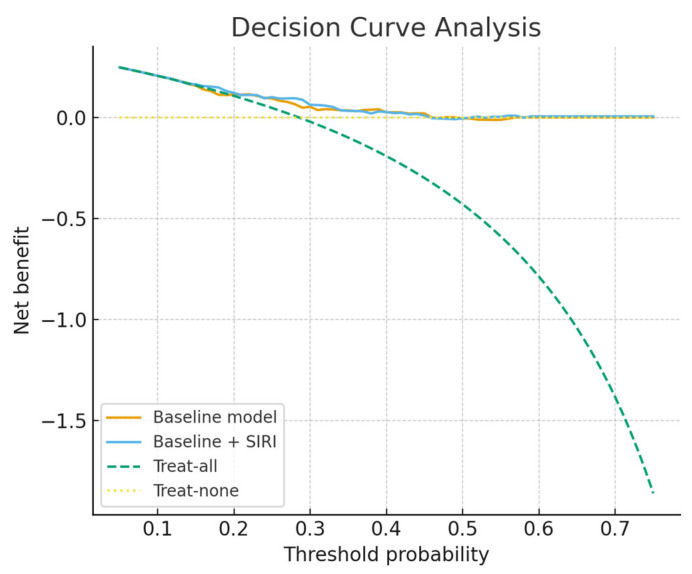
Decision curve analysis of predictive models.

Incremental value analyses further supported the contribution of SIRI, with a statistically significant improvement in integrated discrimination improvement (IDI = 0.024, 95% CI 0.000–0.053, *p* = 0.024) and a positive but non-significant net reclassification improvement (NRI = 0.081, 95% CI −0.031 to 0.193, *p* = 0.086). The comparative performance of the different models is summarised in Table 6. Calibration plots illustrating agreement between predicted and observed probabilities are shown in Figure 4, while Figure 5 presents the decision-curve analysis, which confirmed a higher net clinical benefit for the SIRI-augmented model compared with the baseline model, particularly in the threshold probability range of 0.15–0.45. Calibration-in-the-large demonstrated an intercept of 0.07 (95% CI −0.53 to +0.67) and a slope of 1.08 (95% CI 0.50 to 1.66), indicating good overall calibration without systematic bias or attenuation of predictions. Restricted cubic spline analysis (df = 3) and a Box–Tidwell transformation further confirmed no evidence of non-linearity (LR = 0.23, *p* = 0.97; Box–Tidwell *p* = 0.40), supporting the treatment of SIRI as a continuous linear predictor.

## 4. Discussion

Systemic inflammatory response indices derived from routine blood counts have emerged as potential predictors of postoperative complications across diverse major surgeries [11]. Simple ratios such as NLR, PLR and MLR reflect the balance between innate immune activation and lymphocyte-mediated response and have been widely studied as markers of perioperative inflammatory status [12]. More recently, composite indices like SIRI and AISI have been introduced to capture the overall inflammatory burden [8,13,14]. Numerous studies in oncologic and general surgery settings suggest that elevated pre- or postoperative values of these indices are associated with higher complication rates. For example, in lung cancer patients undergoing thoracic surgery, higher postoperative NLR and MLR were significantly correlated with an increased incidence of surgical complications [12]. Similarly, in gastrointestinal oncology, patients with high preoperative NLR or PLR have shown a greater risk of postoperative morbidities after colorectal cancer resections [15]. Emerging evidence from other major surgical fields further supports this link: a large cohort study in orthopedic surgery reported that patients in the highest quartiles of preoperative SIRI, AISI, and MLR had markedly elevated odds of acute kidney injury after joint replacement—a serious postoperative complication—compared to those with lower inflammatory indices [16]. In urologic oncology, inflammatory markers have also been investigated as prognostic tools; for instance, recent analyses in radical cystectomy for bladder cancer evaluated NLR, PLR, LMR, and related indices as predictors of perioperative complications and 30-day readmissions [17]. While the predictive power of individual markers in that context was modest (one study noted that the preoperative immune-inflammation scores had only limited reliability for forecasting complications) [17], the overall trend in the literature indicates that heightened systemic inflammation tends to portend worse surgical outcomes [18,19]. In summary, systemic inflammatory indices such as SIRI, AISI, MLR—alongside classical ratios like NLR and PLR—have been explored in a range of abdominal, thoracic, and urological surgeries as inexpensive, readily available biomarkers that may help identify patients at greater risk of postoperative complications [12,16].

Although inflammatory markers such as NLR, PLR and MLR have been widely examined in surgical populations [20,21], emerging evidence indicates that composite indices like SIRI may provide stronger prognostic value by combining information from multiple leukocyte lineages into a single metric [8,22]. In gastrointestinal oncology, SIRI has been reported to outperform conventional ratios in predicting postoperative infectious complications and delayed recovery, suggesting it may better capture the cumulative inflammatory burden [8]. Similar associations have been described in thoracic surgery, where elevated preoperative SIRI correlated with higher rates of postoperative morbidity and prolonged hospital stay [23]. Despite this growing evidence, studies focusing on urologic surgery remain scarce, and, to our knowledge, no prior investigation has systematically assessed SIRI, AISI and MLR in the setting of radical cystectomy. By addressing this gap, our study demonstrates that elevated SIRI is independently associated with major postoperative complications and adds to the expanding body of literature supporting composite inflammatory indices as accessible, low-cost tools for perioperative risk stratification.

The superior predictive performance of SIRI compared to traditional markers like NLR or PLR may be attributed to its comprehensive assessment of systemic inflammation. By incorporating neutrophils, monocytes, and lymphocytes into a single index, SIRI better reflects the complex immune dysregulation that occurs postoperatively [24]. This includes both pro-inflammatory responses (mediated by neutrophils and monocytes) and concurrent immunosuppression (reflected by lymphopenia) [25]. Clinically, our findings suggest that SIRI ≥1.545 could serve as a practical threshold for identifying high-risk patients who may benefit from intensified monitoring or preventive measures, while values below this cutoff may help reassure low-risk patients. The high negative predictive value (83.3%) is particularly promising for clinical decision-making. Future studies should focus on validating these thresholds and developing targeted interventions for high-SIRI patients.

SIRI’s greatest strength as a perioperative marker lies in its practicality: it is derived entirely from a standard complete blood count, requiring no additional tests, cost, or infrastructure, which makes it highly amenable to integration into routine preoperative work-ups. In an era where radical cystectomy carries substantial morbidity despite advances in surgical technique and perioperative care, easily obtainable biomarkers that can refine risk assessment are of substantial value. In our study, SIRI showed a notably high negative predictive value (83.3%), suggesting that patients with low preoperative SIRI levels are unlikely to experience major complications. This finding could be clinically meaningful, as it may help clinicians reassure low-risk patients, facilitate faster recovery protocols, and even guide decisions about safe discharge timing. Conversely, a higher preoperative SIRI may serve as an early warning sign, prompting intensified perioperative surveillance, more aggressive infection prophylaxis, or consideration of admission to higher-acuity care settings in the immediate postoperative period. Beyond its individual use, SIRI could also be incorporated into composite risk models alongside demographic factors, comorbidities, and surgical parameters to improve the precision of complication prediction. Our incremental analyses demonstrated that SIRI provided additional prognostic information beyond standard preoperative variables. Although the absolute improvement in discrimination was modest and did not reach statistical significance (ΔAUC = +0.024, 95% CI −0.022 to +0.071, *p* = 0.16), calibration was notably improved, and decision-curve analysis suggested that the SIRI-augmented model offered greater net clinical benefit within the clinically relevant threshold probability range of 0.15–0.45. Moreover, integrated discrimination improvement was statistically significant, while net reclassification improvement was positive but non-significant. Taken together, these findings indicate that SIRI may refine preoperative risk stratification and complement existing clinical models, despite the need for external validation and larger prospective studies before translation into clinical practice. Importantly, beyond modest discrimination, the model demonstrated excellent calibration, with an intercept close to zero and a slope close to one, supporting the reliability of predicted probabilities for clinical use. Linearity checks further confirmed that SIRI could be appropriately modelled as a continuous predictor, which strengthens the robustness of our findings.

Beyond systemic inflammatory indices, recent advances in molecular biomarker strategies, particularly liquid biopsy approaches, are reshaping prognostication in bladder cancer [26]. While liquid biopsy provides direct insights into tumour biology, its implementation is limited by cost and infrastructure demands. In contrast, indices like SIRI offer a universally available, inexpensive tool, and their integration with molecular approaches may ultimately support a more comprehensive framework for personalised perioperative risk stratification. Notably, SIRI was most strongly associated with infectious complications, which supports biological plausibility given the central role of systemic inflammation in infection-related morbidity. If validated prospectively, such an approach might ultimately support a more personalized allocation of perioperative resources—ensuring high-risk patients receive targeted interventions while sparing low-risk patients from unnecessary monitoring or prolonged hospitalization.

This study has several limitations that should be acknowledged. First, its retrospective and single-centre design introduces inherent risks of selection bias and limits the generalisability of the findings to broader patient populations. Second, although we included a relatively homogenous cohort of 200 patients, the sample size may still have constrained the statistical power to detect smaller effect sizes, particularly for secondary inflammatory indices such as AISI and MLR. Third, all patients in this series underwent ileal conduit urinary diversion, which standardises surgical technique but restricts extrapolation of the results to other diversion types such as neobladders. Moreover, our inclusion criteria—patients with TURBT pathology of stage T2 or higher and without prior neoadjuvant therapy—created a more uniform study group but limited the applicability of these findings to patients with different disease stages or treatment histories. Additionally, the analysis focused on complications within 30 days of surgery; while this time frame captures the majority of significant perioperative events, it does not address longer-term outcomes. Another limitation is that some patients experienced more than one type of complication; therefore, subtype analyses were not mutually exclusive. Each subtype was analysed independently, and the findings should be interpreted as exploratory rather than confirmatory. Finally, although we applied bootstrap internal validation, external validation in larger multicentre prospective cohorts is warranted to confirm the reproducibility and generalisability of our findings.

## 5. Conclusions

In conclusion, this study demonstrates that SIRI, a simple and inexpensive biomarker derived from routine blood counts, is independently associated with major complications following radical cystectomy. Among the inflammatory indices evaluated, SIRI showed the strongest predictive performance, with a notably high negative predictive value, suggesting it may help identify patients at low risk for adverse outcomes while flagging those who may benefit from closer perioperative monitoring. While these findings add to the growing body of evidence supporting composite inflammatory indices as prognostic tools in surgical oncology, further prospective, multi-institutional studies are needed to validate SIRI’s predictive utility and to explore its integration into preoperative risk models aimed at optimising patient selection, counselling, and postoperative care.

## Figures and Tables

**Table 1:** Comparison of demographic and clinical variables between patients with and without major complications.

Variable	No Major Complication (*n* = 143)	Major Complication (*n* = 57)	*p*-Value
Age (years)	67.6 ± 7.0	68.4 ± 7.2	0.435
BMI (kg/m^2^)	26.2 ± 4.3	25.6 ± 4.5	0.428
Sex (Male %)	83.20%	85.90%	0.632
Hypertension (%)	49.00%	59.60%	0.132
Diabetes Mellitus (%)	16.80%	29.80%	0.042 *
Coronary Artery Disease (%)	17.50%	26.30%	0.159
Albumin (g/dL)	3.99 ± 0.27	3.96 ± 0.28	0.438
Creatinine (mg/dL	1.09 ± 0.21	1.11 ± 0.15	0.408
eGFR (mL/min/1.73 m^2^)	73.1 ± 16.2	70.9 ± 13.8	0.488
Smoking—Never (%)	75 (52.4%)	35 (61.4%)	
Smoking—Former (%)	37 (25.9%)	12 (21.1%)	
Smoking—Current (%)	31 (21.7%)	10 (17.5%)	0.517
ASA Score = 2 (%)	32 (22.4%)	14 (24.6%)	
ASA Score = 3 (%)	85 (59.4%)	33 (57.9%)	
ASA Score = 4 (%)	26 (18.2%)	10 (17.5%)	0.946

* Statistically significant.

**Table 2:** Distribution of preoperative inflammatory indices (median [IQR]) according to major postoperative complication status.

	No Major Complication (*n* = 143)	Major Complication (*n* = 57)	*p*-Value
SIRI	1.63 [1.11–2.55]	2.12 [1.41–3.35]	0.006 *
AISI	446.9 [270.2–760.1]	487.8 [317.5–956.3]	0.586
MLR	0.30 [0.21–0.42]	0.32 [0.25–0.45]	0.779

* Statistically significant. Values are presented as median (interquartile range). Comparisons were made using the Mann–Whitney U test. SIRI: Systemic Inflammation Response Index; AISI: Aggregate Index of Systemic Inflammation; MLR: Monocyte-to-Lymphocyte Ratio.

**Table 3:** Distribution of preoperative SIRI values according to complication subtypes.

Complication Subtype	*n* (Yes)	*n* (No)	Median SIRI (Yes)	Median SIRI (No)	Mann–Whitney U	*p*-Value	Effect Size (rbc)
Infectious	48	152	1.91	1.41	2632	0.004	0.279
Wound	21	179	1.87	1.49	1379	0.046	0.267
Cardiopulmonary	18	182	1.9	1.5	1288	0.136	0.214
Thrombotic	5	195	1.66	1.52	446	0.748	−0.085
Anastomotic	4	196	1.46	1.52	360	0.78	−0.083

Comparison of preoperative SIRI values between patients with and without specific complication subtypes. Values are presented as medians with Mann–Whitney U test *p*-values and effect sizes (rank biserial correlation).

**Table 4:** Diagnostic performance metrics of the Systemic Inflammation Response Index (SIRI) for predicting major postoperative complications following radical cystectomy.

Metric	Value	95% CI
AUC	0.624	—
Sensitivity	70.20%	56.6–81.6%
Specificity	59.40%	50.9–67.6%
Positive Predictive Value	40.80%	34.7–47.2%
Negative Predictive Value	83.30%	76.7–88.4%
Accuracy	62.50%	55.4–69.2%
Positive Likelihood Ratio	1.73	1.33–2.25
Negative Likelihood Ratio	0.5	0.33–0.76

**Table 5:** Multivariate logistic regression analysis predicting major complications.

Variable	OR (95% CI)	*p*-Value
SIRI	1.37 (1.01–1.86)	0.045 *
Age	1.00 (0.96–1.04)	0.909
BMI	0.93 (0.85–1.01)	0.158
Hypertension	1.65 (0.87–3.11)	0.127
Diabetes Mellitus	1.89 (0.95–3.86)	0.07
Coronary Artery Disease	1.93 (0.94–3.98)	0.093

* Statistically significant. Multivariable logistic regression was performed to identify independent predictors of major postoperative complications (Clavien-Dindo grade ≥ IIIa). No multicollinearity was observed (VIF < 1.1 for all variables). Statistical significance was set at *p* < 0.05. SIRI: Systemic Inflammation Response Index, BMI: Body Mass Index.

**Table 6:** Comparative performance of predictive models for major postoperative complications after radical cystectomy.

Model	AUC (95% CI)	HL χ^2^ (df = 8)	HL *p*-Value	ΔAUC (95% CI)	IDI (95% CI)	NRI (95% CI)
SIRI only	0.624 (0.538–0.709)	–	–	–	–	–
Baseline clinical model	0.648 (0.560–0.735)	10.41	0.236	Ref	–	–
Baseline + SIRI	0.672 (0.586–0.752)	3.33	0.91	+0.024 (−0.022 to +0.071, *p* = 0.16)	0.024 (0.000–0.053, *p* = 0.024)	0.081 (−0.031 to 0.193, *p* = 0.086)

Baseline model includes age, BMI, hypertension, diabetes mellitus, and coronary artery disease. HL = Hosmer–Lemeshow goodness-of-fit test; IDI = Integrated Discrimination Improvement; NRI = Net Reclassification Improvement.

## Data Availability

The datasets generated and/or analysed during the current study are not publicly available due to privacy restrictions, but a de-identified data dictionary and R analysis code are available from the corresponding author on reasonable request.
